# The Role of p38γ in Cancer: From review to outlook

**DOI:** 10.7150/ijbs.63537

**Published:** 2021-09-23

**Authors:** Wentao Xu, Rui Liu, Ying Dai, Shaocheng Hong, Huke Dong, Hua Wang

**Affiliations:** 1 Department of Oncology, the First Affiliated Hospital of Anhui Medical University, Hefei, 230022, Anhui, China.; 2 First Clinical Medical College of Anhui Medical University, Hefei, 230032, Anhui, China.; 3 Inflammation and Immune Mediated Diseases Laboratory of Anhui Province, Anhui Medical University, Hefei, 230032, Anhui, China.

**Keywords:** p38γ, molecular pathway, intestinal microbes, gene editing, immunotherapy

## Abstract

p38γ is a member of the p38 Mitogen Activated Protein Kinases (p38 MAPKs). It contains four subtypes in mammalian cells encoded by different genes including p38α (MAPK14), p38β (MAPK11), p38γ (MAPK12), and p38δ (MAPK13). Recent studies revealed that p38γ may exhibit a crucial role in tumorigenesis and cancer aggressiveness. Despite the large number of published literatures, further researches are demanded to clarify its role in cancer development, the tissue-specific function and associated novel treatment strategies. In this article, we provide the latest view on the connection between p38γ and malignant tumors, highlighting the function of p38γ. The clinical value of p38γ is also discussed, helping the translation into the remarkable therapeutic strategy in tumor diseases.

## Introduction

To date, the p38γ, the third mammalian p38, is initially described as a novel protein kinase from rats 1, known to assist local phosphorylation in the cell nucleus 2. p38γ is a 43-kDa 3 and 367-amino acid protein 4 with a characteristic C-terminal motif KETXL. It can be docked with the PSD-95/Dlg/ZO-1 homology (PDZ) domain of additional proteins, such as α 1-syntrophin, PTPN3, SAP90/PSD95 and the scaffold protein SAP97/hDlg. p38γ binds its PDZ domain of these proteins and induces the phosphorylation 5, 6. For instance, the environmental osmotic pressure changes trigger p38γ activation in the cytoplasm and concentrate in the nucleus, thereby increasing the binding of p38γ and nuclear hDlg and resultantly disassembly of the hDlg-PSF (protein-associated-splicing factor) complex 7, 8. Due to its capacity to shuttle between cytoplasm and nucleus, p38γ might establish a link between two key procedures of gene expression and cytoskeleton reorganization to adapt to environmental changes.

Notably, p38γ can be activated by the stress and mitogenic signals, and further promote tumorigenesis and progression of cancer 9. For example, p38γ is activated by Ras and is indispensable for Ras transformation and invasion 2, 10, 11. p38γ is located predominantly in the nucleus 3. Meanwhile p38γ is capable of inhibiting cell migration, essential for oncogene-induced senescence 12. These findings indicate that p38γ probably has tumor suppressive or carcinogenic effects (Table 1).

## Overview of MAPKs

Mitogen-activated protein kinases (MAPKs) are serine/threonine protein kinases that convert extracellular stimuli into a variety of cellular responses 13. The RAS/RAF/MEK/ERK (MAPK) signaling cascade, one of the widespread signaling pathways, plays a vital role in cellular processes whose cell disorders relevant to carcinogenesis and cancer development, including differentiation, propagation, inflammation, apoptosis, survival and innate immunity 13. In the early 80s, ERK1 appeared in the focus as the first MAPK core molecule to be discovered and identified in mammals 14. Subsequently, the highly homologous ERK2 and ERK3 were cloned one after another, and the research on the mammalian MAPK pathway began 14. Currently, typical MAPKs mainly include ERK1/2, JNK1/2/3, p38 isoforms accompanied by in-depth studies 14. Furthermore, each classic MAPK consists of a set of three kinases acting in sequence: a MAPK, a MAPK kinase (MAPKK) and a MAPKK kinase (MAPKKK), and the extensive functions they regulate are principally mediated by phosphorylation of members of the protein kinase family named MAPK-activated protein kinases (MAPKAPKs) 14.

## Overview of p38

The p38 MAPK family is one of the four major mitogen-activated protein kinases (MAPKs) cascades as a class of highly conserved proteins found firstly in studies of endotoxin-induced cytokine expression 10, 15-17. It was reported to get involved in inflammation, cell growth, cell differentiation, cell death, and the cell cycle 10, 15, 18-21. So far, there are four p38 isoforms respectively named p38α (MAPK14), p38β (MAPK11), p38γ (SAPK3, ERK6 or MAPK12), and p38δ (MAPK13) 5. These kinases share extremely analogical protein sequences; p38α and p38β have 75% identity, whereas p38γ and p38δ have 62% and 61% identity with p38α, respectively. Simultaneously, p38γ and p38δ have 70% similarity with each other 22.

## p38γ and Cell cycle

Targetedly, cells are capable to dispose DNA damage by terminating the cell cycle process or performing procedural cell death 23. Highly conservative DNA repair and cell cycle checkpoint pathways enable cells to cope with endogenous and exogenous DNA damage 23.

**Figure 1:**
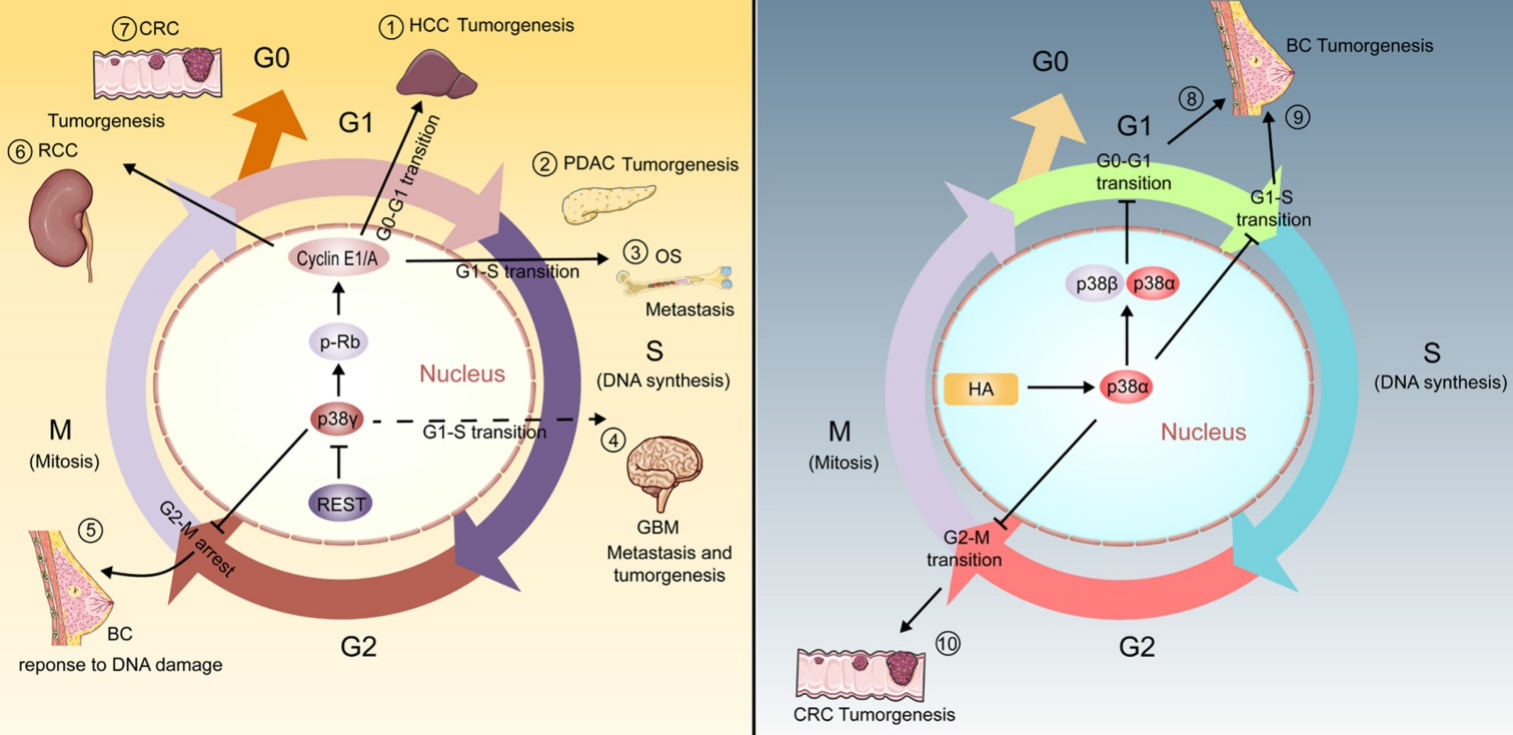
** p38γ participates in the biological process of various cancers by regulating the cell cycle process.** (1) p38γ/p-Rb/Cyclin (E1/A) pathway could encourage tumorigenesis and proliferation of hepatocellular carcinoma by promoting G0-G1 phase transition; (2) p38γ/p-Rb/Cyclin (E1/A) pathway could promote the transition of the cell cycle from G1 to S phase, and is affiliated to tumorigenesis, proliferation, and motility of pancreatic ductal carcinoma; (3) p38γ/p-Rb/Cyclin (E1/A) pathway could promote the transition of the cell cycle from G1 to S phase, connected with tumorigenesis, proliferation, and invasion of osteosarcoma; (4) As a downstream target of transcription factor REST, p38γ can be restrained by REST and may promote the transition of G1-S phase, resulting in the proliferation and migration of glioblastoma; (5) p38γ causes G2-M phase arrest and maintains breast cancer cell survival under conditions of DNA damage; (6) p38γ/p-Rb/Cyclin (E1/A) pathway may play a crucial part in the proliferation, development and migration of human renal cell carcinoma cells; (7) p38γ/p-Rb/Cyclin (E1/A) pathway may play a vital role in the proliferation, development and invasion of human colorectal cancer cells; (8) Hyaluronan-dependent p38α/β activation induces GO-G1 phase arrest, leading to growth inhibition of breast cancer; (9) Blocking G1-S phase conduction by p38α also inhibits the tumorigenesis of breast cancer; (10) p38α blocks G2-M phase transmission and weakens the tumorigenesis of colorectal cancer.

In the past decade, p38 pathway has been emphasized as a core part of cell cycle regulator in cancer which principally manifested as the effect of p38α/β/γ instead of p38δ (Figure 1). For example, in human breast cancer MDA-MB-231BO cells, hyaluronan activates p38α/β and up-regulates the expression of p21^cip1^, leading to the decrease of Cyclin D1 level, prolonging the conduction in G0-G1 phase and slowing the growth of breast cancer cells 24. p38α can also phosphorylate Rb, down-regulate the expression of E2F transcription factor, delay the progression of G1-S phase and reduce tumor cell proliferation in MCF7 breast cancer cells 25. Likewise, the activation of p38α-dependent p53 promotes the expression of GADD45α, inhibits the activity of the Cdc2/Cyclin B1 complex, and achieves the function of inhibiting the growth of HCT116 human colorectal carcinoma cells by blocking the G2-M phase 26. A study has reported that circTNFRSF21 is the upstream of p38δ, and its expression enhancement can promote the transition of endometrial carcinoma cells from G0/G1 to S phase. However, the mechanism by which p38δ regulates the cell cycle is still unclear 27.

As early as 1999, p38γ has been found to inhibit cell proliferation by down-regulating cyclin D1 during adrenal cell hypoxia 28. Moreover, it is proved that the activation of ATM/MKK6/p38γ/Cds1 pathway is essential for the correct regulation of G2 checkpoints in mammalian cells 29. In line with this, it has been proved that Xenopus p38γ can promote the meiosis of Xenopus oocytes treated with progesterone, while p38α or p38β have no effect 30. On this basis, fully grown Xenopus oocytes are blocked in prophase of meiosis I and are induced to pass through meiosis by progesterone stimulation 30. In human cells, it has also been found that the presence of p38γ can cause cell cycle changes, and this may be essential for the proliferation, invasion and carcinogenic activity of numerous cancers 16, 31. Similarly, the ectopic expression of p38γ may contribute to a significant arrest of the cell cycle in G2/M phase, which may be a mechanism of breast cancer cells to survive established on DNA damage 32, 33.

Taken together, p38γ participates in the regulation of the cell cycle, contributing to a variety of cancers 16, 31, 32, 34-37 (Figure 1). Considering the prominent position of p38γ in regulating the cell cycle process, the role of p38γ in cell cycle regulation in more cancers is worthy of further investigation.

## Breast Cancer

Estrogen receptor (ER) is a transcription factor related to BC, which has the paradoxical effect of promoting proliferation and resisting invasion 11, 38, 39. It has been reported that p38γ can integrate the invasive antagonism of Ras and ER without affecting the proliferative activity of BC, thereby promoting the invasiveness of BC 11. Involvement in cellular metabolism is another mechanism by which p38γ increases BC invasiveness. Using a novel metabolomics approach to demonstrate the role of p38γ in apoptosis, invasion, and migration, Chen et al. showed the potential to promote cancer metastasis, particularly altering the enrichment of certain metabolites and metabolic pathways, which may bring potential to the evolution of novel therapeutic strategies for BC 40.

Another study based on a particular kinetic cell movement analysis method further certifies that p38γ can control cell movement by regulating the metastasis-associated small GTPase-RhoC ubiquitination and lysosomal degradation, as well as regulate diversifications in cell structure and cell form of BC 41. This evidence suggests p38γ may possess a fundamental effect in the metastatic properties of BC. Apart from that, there is pertinence between enhancive expression of p38γ and the decline in the overall survival rate of BC patients 41.

In ethanol-induced mice, loss of p38γ can alleviate BC development and metastasis, and knockdown of p38γ can block alcohol-aroused RhoC activation, cell spread, invasion and migration 42. Correspondingly, there is a significant positive correlation between the expression of p38γ and RhoC in clinical BC samples 41. Alcohol has also been reported to augment the invasiveness of BC cells in another study. It stimulates the phosphorylation of p38γ by activating ErbB2 and promotes the interaction of p38γ with the substrate SAP97/DLG as well as the phosphorylation of SAP97/DLG, thus leading to the activation of CSCs (cancer stem cells) 43.

Epithelial cell-mesenchymal transition (EMT) refers to the biological process of epithelial cells transforming into cells with mesenchymal phenotype through specific procedures and helps cancer cells spread from solid tumors and form detectable metastasis 44. It was previously shown that p38γ could facilitate EMT and CSCs in BC cells, as evidenced by reductive E-cadherin expression and incremental Vimentin level 45. In this course, ErbB2 and ErbB4 act as the major upstream receptor kinases. Both are capable to activate p38γ through ubiquitin-proteasome-dependent degradation and the degradation of GATA3 is caused subsequently. This suppression causes the up-regulation of miR-200b and down-regulates the expression of Suz12, eventually leading to EMT and invasiveness of BC 45.

Distinguishingly, among all types of BC, triple negative BC (TNBC) tends to have the worst prognosis due to the lack of therapeutic targeting of ER, progesterone receptor (PR), and epidermal growth factor receptor 2 (HER-2) 46. Meanwhile TNBC is prominently heterogeneous but abundant among the CSCs population 47. Qi et al. point out p38γ activates Nanog transcription by forming a polyprotein complex with c-Jun/Hsp90 on the Nanog promoter and activates the expression of transcription factors such as Sox2 and Oct3/4 to stimulate CSCs amplification in TNBC cells. These consequences further suggest that the coalescence of Hsp90 inhibitors with the non-toxic p38γ inhibitor PFD may even be more efficacious in treating TNBC by destroying functional protein complexes, thereby co-consuming the CSCs population 48.

## Squamous Cell Carcinoma

Lack of p38γ makes the incidence and number of tumors per mouse lower than that of wild-type mice 21. Researchers demonstrated that p38γ and p38δ are concerned with the production of cytokines such as IL-6, STAT3, and TNF-α in the skin, contributing to skin tumor evolution. The loss of p38γ versus p38δ abrogated tumorigenesis in human epidermoid carcinoma A431 cells 21.

In the case of esophageal squamous cell carcinoma (ESCC), stable down-regulation of p38γ can prevent tumorigenesis in a nude mouse model of ESCC cell xenotransplantation 49. In addition, p38γ can promote the movement and proliferation, and prevent the apoptosis of ESCC cells *in vitro* 49. Similarly, higher p38γ expression is also observed in head and neck squamous cell carcinoma cells 20.

## Hepatocellular Carcinoma

p38γ is immediately related to the expression of ACTA2 and COL1A 31. Both these two genes encode actin and collagen respectively, which are fibrotic markers that commonly used before liver cancer develops, indicating the HCC with p38γ elevation is easier to migrate and deteriorate 31.

Experiments by Tomas-Loba et al. showed that p38γ staining in human HCC biopsy tissues is much stronger than in non-tumor tissues and p38γ knockdown weakened proliferation and colonization in HCC cell lines 31. In addition, HCC in mice lacking p38γ in hepatocytes (AlbCre-p38γ mice) is observed to be strongly controlled. Compared with control (AlbCre+/-) mice, their tumors were smaller, fewer, and survived longer 31. Albcre-p38γ mice additionally showed the ability to prevent liver cancer induced by carbon tetrachloride or high-fat diet 50. As a cyclin-dependent kinase (CDK)-like kinase, p38γ exhibits a sort of substrate specificity and inhibition sensitivity analogical to conventional CDKs, which is manifested as phosphorylate the C terminus of retinoblastoma tumor suppressor protein (Rb) to promote liver proliferation 16, 31. p38γ may stand for a unique kinase that activates the non-classical CDK pathway in a cell cycle protein-independent manner when the CDK-cyclin complex is low, thus initiating the cell cycle and allowing the cells to escape from quiescence under stress stimulation. Collectively, these evidences undoubtedly confirm that p38γ is necessary for Rb-dependent cell cycle progression and liver tumorigenesis, supporting the potential of p38γ as a target for HCC treatment.

## Colorectal Cancer

Loss of p38γ significantly inhibits cell growth, proliferation, and migration in HT3-29 cells and primary human CRC cells, while suppressing apoptosis. In particular, p38γ standards are observably increased in human CRC tissues in terms of the surrounding normal epithelial tissues 34. Consistent with previous research, p38γ deletion also results in decreased Rb phosphorylation and cyclin (E1/A) expression, both of which are augmented as p38γ highly expressed in CRC cells 34.

As an important regulatory factor, p38γ promotes CRC tumorigenesis by transmitting exogenous and endogenous signals. For example, conditional p38γ knockout of intestinal epithelial cells (IECs) reduces the expression of proinflammatory cytokine and β-Catenin/Wnt pathway activity in colon tissues, successfully linking inflammation with CRC 51. Chronic application of PFD can alleviate CRC induced by inflammation in mice, but no effect in mice with conditional p38γ knockout in IECs 51. In addition, in the AOM/DSS mouse model, p38γ deficiency also attenuates the generation of CRC 51.

The Ras-dependent mechanism is an important part of the occurrence of CRC 52. PTPH1 acts as an exclusive p38 subtype-specific phosphatase known to interact through PDZ binding 53. Hou et al. found through experiments that p38γ can directly phosphorylate PTPH1 to promote the growth of Ras conversion and Ras-dependent CRC 52. In human CRC tissues, the transcription factor c-Jun was activated by phosphorylated p38γ through its C-terminus, and then the activated c-Jun recruited p38γ as a cofactor to the matrix metalloproteinase 9 (MMP9) promoter, inducing its transactivation and ultimately promoting CRC cell invasiveness 3.

## Pancreatic Ductal Adenocarcinoma

p38γ has been reported to link K-Ras signaling and the Warburg effect by means of phosphofructokinase-2/fructose-2,6-bisphosphatase 3 (PFKFB3) and glucose transporter 2 (GLUT2). It's crucial for aerobic glycolysis and pancreatic tumorigenesis 16. The p38γ activation-dependent phosphorylation and stabilization of PFKFB3 and its interaction with GLUT2 constitute a ternary complex, which can effectively boost the occurrence of aerobic enzymes and pancreatic ductal adenocarcinoma (PDAC) via their regionally elevated concentrations. The course of cell cycles from the G1 to S phase in p38γ silenced pancreatic ductal epithelial cells (KPC) cells has been found to be blocked compared with KPC and associated with lower levels of CDK6 (cyclin dependent kinase 6) and p-Rb proteins 16. The function of p38γ in the G1/S transition may be critical for stimulating cell proliferation and cell motility, activating glycolysis, promoting inflammatory responses as well as the development and growth of PADC 16, 54. In addition, a novel therapeutic strategy established by PFD and the PFKFB3 inhibitor PFK15 can inhibit glucose uptake, reduce lactic acid secretion, and suppress PDAC growth 16.

## Glioma

p38γ defect results in lessened proliferation and enhancive apoptosis of glioma cells 55. With the decrease of p38γ, the expression of human telomerase reverse transcriptase (hTERT) and telomerase show a corresponding decline, while the expression of Caspase-3/9 increases 55. Glioblastoma (GBM) is the most malignant and common glioma 56. Previous studies have shown that the transcription factor REST (repressor element 1-silencing transcription factor) may be present as a cancer gene in GBM 37. As a downstream gene of REST, p38γ can be inhibited by the complex formed by REST and its cofactors, which may trigger the transition of G1-S phase in cell cycle. The inhibition of p38γ by REST may eventually lead to the proliferation and migration of GBM cells, which partially proves the tumor suppressor effect of p38γ 37. In glioma of the entire nervous system, the controversial results shown by p38γ so far may be due to different experimental conditions and experimental designs. Therefore, appropriate experiments are needed to verify the fundamental role of p38γ in glioma next.

## Cutaneous T-cell Lymphoma

p38γ is likely to be an important breakthrough in improving the level of CTCL diagnosis and treatment in the future. It selectively increases its expression to promote cell activity in CTCL patients and cell lines 4. Meanwhile multi-kinase inhibitor F7/PIK75 could efficaciously restrain p38γ enzymatic activity and the production of its phosphorylated substrate pDLDH1 in CTCL cells and mouse xenografts 4.

Sézary syndrome (SS) is a form of CTCL that appears on the skin. Based on different investigations *in vitro*, for instance, the expression of p38γ is higher in CD^+^T cells in patients with Sézary syndrome than in healthy blood donors 57. In 2018, a private non-profit clinical research center and hospital called City of Hope filed for a patent, which included a new p38γ inhibitor and its application in the treatment of CTCL 57. The lead compound inhibitor in this patent has ATP competitive inhibitory effect on p38γ, and this compound shows dose-dependent inhibitory effect on tumor growth in CTCL xenograft model 57. The additional library of EMD Biosciences screens 260 kinase inhibitors, including two novel potential p38γ inhibitors, and shows a typical-I p38 inhibitor binding schema to determine that p38γ is a potential objective for the therapy of CTCL 57.

## Osteosarcoma

miR-187 is confirmed to be the direct upstream molecule of p38γ in osteosarcoma cells 58. The low level of miR-187 is significantly related to staging, lymph node metastasis and deep stromal infiltration. By inhibiting p38γ, it reduces the biological behaviors of osteosarcoma cells like cell proliferation, migration and invasion. Correspondingly the reverse expression of miR-187 and p38γ in specimens of OS patients further confirmed this process 58.

Notably, the main cause of OS abnormal growth is the uncontrolled cell cycle and cell division 59. p38γ can also play a cancer-promoting part in OS by stimulating Rb phosphorylation and cyclin E1/A expression and regulating G1-S phase transition 36. Accordingly, present study shows that mRNA and protein expression of p38γ in human OS tissue and primary OS cells were significantly higher than that in human primary osteoblasts and OB-6 osteoblastic cells 36.

## Renal Cell Carcinoma

p38γ can analogously serve as a novel cyclin dependent kinase (CDK)-like kinase, which can phosphorylate Rb at the molecular level and regulate the cell cycle protein cyclin E1/A expression, ultimately promoting the progression of RCC cells 35. Correspondingly, ectopic overexpression of p38γ promoted the growth, proliferation and migration of 786-O cells and primary RCC cells 35. Besides, it manifested an increase of p38γ mRNA and protein degree in human renal cell carcinoma instead of adjacent tissues 35. The results of Aguilar et al. showed that the level of p38γ in the kidney increased gradually after 1 and 2 months of cancer induction with ferric nitrilotriacetate (FeNTA) 60. In this case, with the evolvement of cancer, the level of p38γ in the tumor even reached more than 30 times of that in the normal kidney 60.

## Non-Small Cell Lung Cancer

Cancer stem cells (CSCs) are the main source of tumorigenesis and recurrence and participate in p38γ-mediated inhibition of NSCLC. Activated p38γ can mediate the ubiquitination and degradation of stem cell proteins such as Sox2, Oct4 and Nanog by means of activating the downstream kinase MK2 to phosphorylate Hsp27, thereby inhibiting the tumor stem cell characteristics and tumor initiation ability of NSCLC cells 12. Conversely, a significant increase in p38γ is associated with carcinogenicity of NSCLC, but not with stage, tumor, or response to treatment 61. As of yet, the research of p38γ in NSCLC is limited, especially when the current opinions are contradictory without the specific role and mechanism of p38γ.

## Bladder Cancer

**Figure 2:**
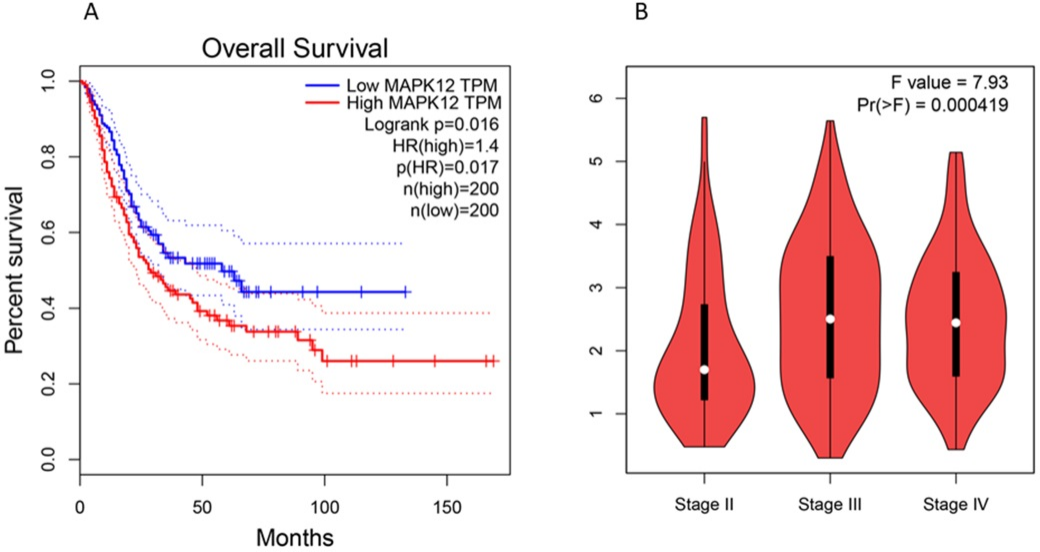
** Expression of p38γ in different survival rates and different stages of bladder cancer.** (A) Correlation analysis between p38γ and overall survival in bladder cancer (p = 0.017); (B) The expression of p38γ in different clinical stages of bladder cancer (p = 0.000419).

Kaplan Meier analysis shows that the expression of p38γ in bladder cancer is significantly different (P < 0.05), and the prognosis of patients with high expression of p38γ is often gravely unfavorable 62. p38γ may have the ability to become a prognostic marker and therapeutic target of bladder cancer. We used GEPIA (Gene Expression Profile Interactive Analysis): http://gepia.cancer-pku.cn/ to conduct a correlation analysis and found that the high expression of p38γ in bladder cancer was negatively bound up with the overall survival of patients. Additionally, p38γ expression in different pathological stages (stage II, stage III, and stage IV) of bladder cancer had conspicuous significance (Figure 2). However, the mechanism of p38γ in bladder cancer is still obscure in experiments, consequently p38γ may be used as a potential research target.

## The latent clinical value of p38γ in human carcinoma

The early detection and diagnosis of cancer is crucial for patients. Sensitive tumor biomarkers are capable of reflecting the molecular differences of certain exceptive cancers when they occur at an early stage, which is not only propitious to inchoate diagnosis, but also advantageous to the screener and prognostic judgment of synthetical treatment tactics. Coincidentally, relevant studies have shown that the expression of p38γ is closely bound up with the depth of invasion, clinical stage, metastasis, and chemosensitivity. This section summarizes reports of p38γ as a biological marker for cancer diagnosis and prognosis and also summarizes the effect of p38γ on the sensitivity of tumor chemotherapy.

Literatures have demonstrated the value of p38γ in diagnosis. For example, researchers use immunohistochemistry with tissue microarray and performed a statistical analysis of its clinicopathological significance 49. The expression of p38γ in ESCC is significantly different between clinical stage (P = 0.017), lymph node metastasis (P = 0.008) and tumor volume (P = 0.017) 49. In terms of prognosis, no conspicuous discrepancy is observed between patients with different levels of p38γ expression (P = 0.667) in ESCC 49. In a HCC cohort composed of 372 HCC patients, the Kaplan-Meier survival curve of patients stratifies by p38γ expression shows that p38γ overexpression is associated with poor prognosis of HCC patients 31. Among 43 samples of BC patients whose molecular subtypes are analyzed by PAM50 before, high expression of p38γ is accompanied by the obvious enrichment of BC basal-like subtypes (P = 0.018) 41. The expression of p38γ is also analyzed in 118 BC patients, including survival data. The results show that there is a consanguineous correlation between the higher expression of p38γ and the lower overall survival rate (P = 0.013), suggesting that the expression of p38γ is of great significance for the prognosis of BC patients 41.

Chemotherapy still consists of the mainstay in cancer treatment. It has been reported that p38γ can transduce signals related to three widely used chemotherapy drugs, cisplatin, etoposide and tamoxifen 63.

## Outlook

A serry of studies showed the impact of p38γ on carcinogenesis, invasion, maintenance and resistance to chemotherapy. The p38γ signal pathway leads to incremental expression of multiple downstream targets including transcriptional regulators. The transcription targets regulated by p38γ may be either oncogenes or tumor suppressors, determined by different organs and tissue. Among the associated research of p38γ in cancer, conflicting data in specific cancer types indicate that the full role of p38γ is unclear, and further research is required to adequately comprehend the ponderance of p38γ in human pathology. This section summarizes the update and the prospect of p38γ in life sciences.

Drugs that directly target DNA topoisomerase II α (Topo IIα or Topo II) represent a vital class of anti-tumor drugs in clinic. It has been proved that p38γ can actively regulate the signal transduction of the drug-Topo II and can improve the therapeutic activity of Topo II drugs 9. In this process, p38γ is specifically activated by Topo II drugs, and thereafter activated p38γ phosphorylates Topo II at Ser-1524, resulting in stronger stability and growth inhibition. The combination of p38γ and Topo II drugs may have clinical value for tumor disease. So far, one p38γ inhibitor PFD was applied in clinical. It has been experimentally proven *in vivo* that its inhibitory effect on p38γ requires a high daily dose (500 mg/kg/day in drinking water) 51, which raises doubts about its clinical value in anti-cancer fields. Conversely, among more than 20 p38 family candidates in clinical trials 57, for instance, combined p38γ and p38δ inhibitor (BIRB796) against IFN-γ 64, multi-kinase inhibitor F7/PIK75 which effectively against p38γ for CTCL treatment 4, pirfenidone is the exclusive drug targeting p38γ clinically for anti-fibrosis 16. Additionally, the Drugbank platform showed that the current experimental and investigational drugs targeting p38γ include Phosphonothreonine, Phosphoaminophosphonic Acid-Adenylate Ester, CEP-1347 and KC706 65. A number of pre-clinical and clinical trials were needed to explore more effective p38γ inhibitors.

The relationship between intestinal microbes and cancer is attracting immense attention in gastrointestinal cancer and cancers of other metabolic organs 66-68. Imidazole propionate is a histidine-derived metabolite produced by microorganisms. In 2018, research by Ara Koh et al. showed that imidazole propionate could activate p38γ to promote the phosphorylation of p62 and activate the mechanical target of rapamycin complex 1 (mTORC1). The demonstration of this impairs insulin signal transduction at the level of insulin receptor substrates. This interaction between the intestinal flora metabolites and p38γ will eventually lead to the development of metabolic diseases, and potentially linked to the pathological conditions of cancer caused by the continuous activation of mTORC1 68. On the other hand, the signal adapter p62 is a multi-domain protein concerned with the activation of the transcription factor NF-κB; the activity of p62 is closely related to the external apoptosis pathway, as well as autophagy, a key factor in tumorigenesis 69. In 2020, further research by Ara Koh et al. found that the microbial metabolite imidazole propionate can reduce the effectiveness of metformin by interacting with the p38γ/AKT/AMPK metformin signaling pathway 70. Although metformin is a major impactful drug for the treatment of type 2 diabetes, epidemiological studies have demonstrated a correlation between the application of metformin and the beneficial effects of cancer therapy 71. In summary, p38γ may play as a key molecule in microbial metabolism that affects the effect of metformin on cancer. We hypothesize that the intestinal flora may regulate the pathogenesis of cancer by influencing p38γ.

Given that cancer is a genetic disease, gene therapy is expected to fight cancer from within with minimal side effects, and there is evidence that CRISPR-Cas9 can correct cancer-causing genome aberrations and induce the expression of tumor suppressor genes to achieve cancer gene therapy 72. Using CRISPR-Cas9 inhibits oncogenes such as p38γ gene in some tumors, which may become a promising anti-cancer strategy. Researchers have used CRISPR-Cas9-induced p38γ knockout to inhibit the *in vitro* progression of human OS cells and the growth, proliferation and migration of human CRC cells and human RCC cells and induce significant apoptosis 34-36. On the other hand, it is reported that CRISPR-Cas9 can be used as a powerful tool to manipulate epigene regulation to treat cancer and reshape abnormal epigenetic patterns and can selectively and genetically alter gene expression 73. For example, nuclease-inactivated dCas9 is coalesced with certain epigenetic modifiers such as acetyltransferases to shape a compound, which enters the target site under the lead of a single guide RNA (sgRNA), and finally exerts an epigenetic regulation effect. This process is also known as epigenome editing 74, 75. p38γ serves as the part of the corresponding gene expression product of epigenetic modification, may also be adaptive for epigenome editing therapy.

Immunotherapy has become a considerable part that cannot be ignored, including immunosuppressants targeting PD-1 for T cells and PD-L1 for tumor cells in recent years. The most cutting-edge Car-T therapy is also getting more attention. The efficacy of immunotherapy varied in individual patients and cancer diseases. The level of PD-L1 is the most influential factor to affect the anti-tumor effect of immunotherapy. Immune checkpoint inhibitors (ICIs) targeting PD-1 or PD-L1 have already greatly improved the prognosis in different cancer types and prolong the patients' survival as well as quality of life. The approach that uses a panel of biomarkers to guide patient selection may provide an opportunity to improve patient outcomes relative to a single biomarker such as PD-L1 76. Based on the studies above, it may be feasible to combine the ICIs prediction biomarker represented by PD-L1 with p38γ.

One of the focus of anti-tumor immunotherapy is to activate CD8^+^T cells and maximize the function of CD8^+^T cells. CD8^+^T cells play a tumor-specific killing effect with the assistance of CD4^+^T cells. As CD8^+^T cells continues to function, immune checkpoints gradually appear on the surface of cell membranes, and bind to receptors from cancer cells, which eventually leads to the death of CD8^+^T cells. This is an immunosuppressive process and is also a major cause of immune escape from tumors. p38γ plays a basilic role in the development and differentiation of T lymphocytes. The deletion of p38γ can facilitate the positive selection of thymocytes from CD4^+^CD8^+^ double-positive cells to CD4^+^ or CD8^+^ single-positive cells 77. Therefore, the clinical use of p38γ inhibitors may coalesce T cell immunotherapy with p38γ itself as an oncogene to develop more effective anti-cancer methods.

## Conclusion

**Figure 3:**
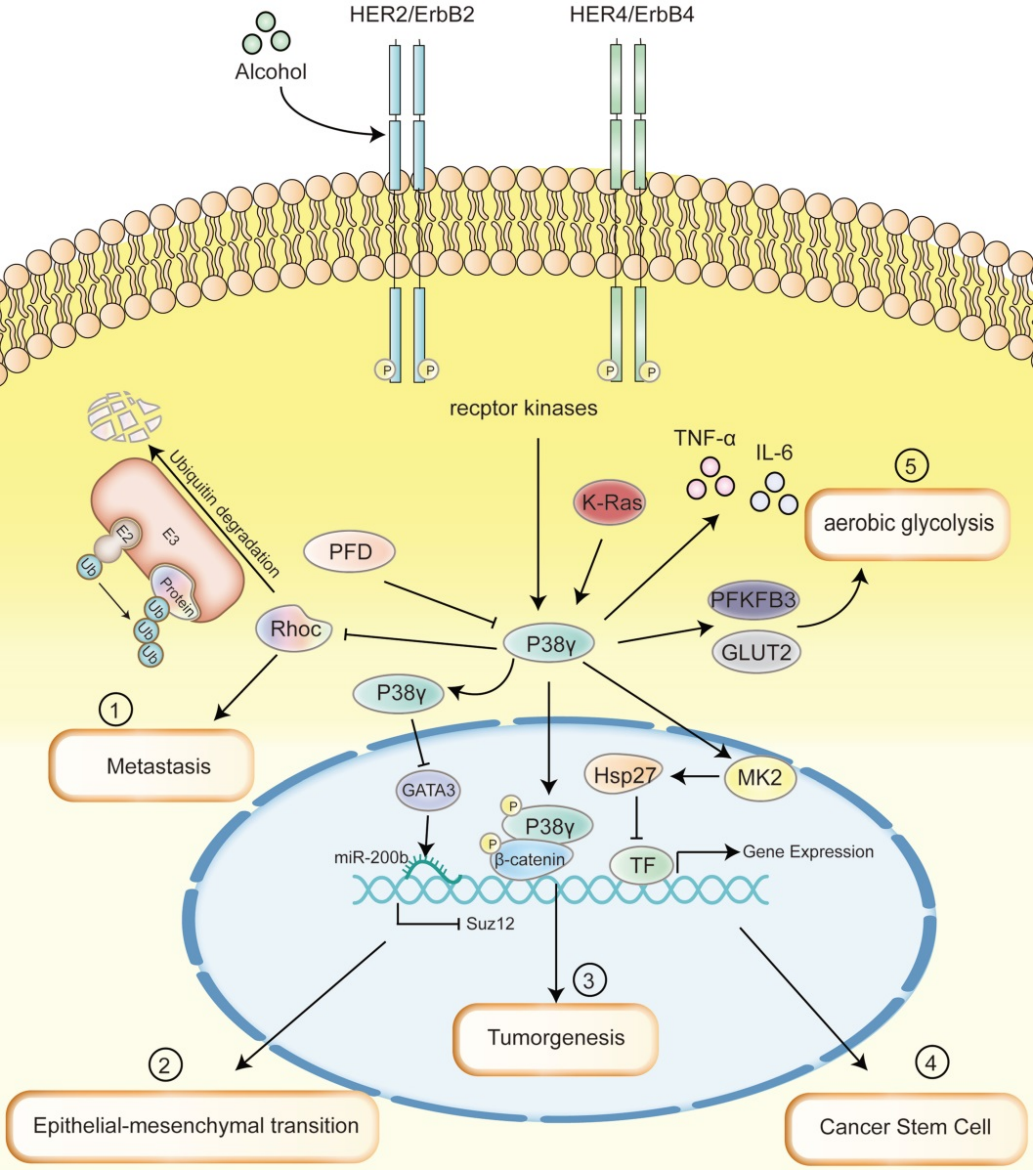
** Multiple signaling pathways that p38γ mainly involved in a range of cancer diseases.** (1) p38γ regulates the ubiquitination of RhoC related to metastasis to control cell movement and promote the metastasis of BC. (2) ErbB2/ErbB4/p38γ/GATA3/miR-200b/Suz12 pathway promotes the acquisition of the EMT phenotype, which may mediate the aggressiveness of BC. (3) The silencing of p38γ in intestinal epithelial cells can reduce the expression of pro-inflammatory cytokines and the activity of β-Catenin/Wnt pathway in colon tissue, thereby successfully linking inflammation with CRC, leading to tumorigenesis of CRC. (4) Upregulated p38γ can phosphorylate Hsp27 protein by activating the downstream kinase MK2, which binds to transcription factor proteins such as Sox2, Oct4, and Nanog to cause ubiquitination and degradation, thereby inhibiting the cancer stem cell characteristics and tumor initiating ability of NSCLC. (5) As the upstream of p38γ, K-Ras promotes the activation of p38γ, increases the expression of PFKFB3 and GLUT2, thus encourages aerobic glycolysis and facilitates the occurrence of PDAC. Throughout the process, cytokines such as TNF-α and IL-6 produced by p38γ link the inflammation and cancer; PFD is able to specifically inhibit the phosphorylation of p38γ.

While the past decade has witnessed the rapid development of cancer researches, cancer treatment methods are nothing new. Ample evidence proved that p38γ possesses a basilic function in the oncogenesis and cancer development, including proliferation, EMT, invasion, metastasis. This heralds that p38γ may play an excellent function in diagnosis, treatment, and prognostic indicators in related tumor types. Despite the role of p38γ inhibitors in clinical tumors is not prominent, there is plenty of room for perfection. We summarized the primary impact of p38γ at different stages of a variety of tumors through several classic signal transduction pathways (Figure 3), besides foremost expectations. The future research orientation of p38γ may be based on large-scale clinical trials and further in-depth mechanisms. p38γ will definitely elicit audacious threads for comprehending and treating cancer.

## Figures and Tables

**Table 1:** The carcinogenic and anticancer effects of p38γ

Type	Observation	Species	References
**Tumors promoted by p38γ signaling**			
BC	p38γ is related to the aggressiveness and metastasis of BC	Human	11, 40,41, 42,43,45
	Alcohol selectively stimulates phosphorylation of p38γ in breast cancer cells	Mouse	43
	Increased p38γ expression is related to a decrease in overall survival rate of BC patients	Human	41
	PFD acts as a targeted inhibitor of p38γ and can inhibit the development of TNBC in cells and/or mice	Mouse	48
SCC	SCC incidence and tumor lesion number per mouse were lower in p38γ- mice than that in WT mice	Mouse	21
	There is a conspicuous correlation between the expression of p38γ and the clinical stage of tumor, tumor volume and lymph node metastasis in ESSC	Human	49
	The expression extent of p38γ is related to HNSCC	Human	20
HCC	In HCC cases, the excessive expression of p38γ is highly indicative of its carcinogenicity and unfavorable prognosis	Human	31
	In mice with absent p38γ, HCC is strongly inhibited, and survival time is prolonged	Mouse	31
	p38γ inhibitor PFD is able to prevent the occurrence of HCC induced by DEN	Mouse	31
CRC	Highly expressed p38γ boosts the growth, proliferation, cell migration and apoptosis resistance of CRC cells in human CRC tissues	Human	34
	Mice with IEC-specific p38γ deletion in the AOM/DSS model have reduced colon tumor formation	Mouse	51
PDAC	p38γ is involved in the spread of PDAC and aggressive behavior	Mouse	16
	The pharmacological p38γ inhibitor PFD inhibits carcinogenesis effect and tumor growth in PDAC	Mouse	16
Glioma	p38γ is related to the proliferation and apoptosis of glioma, and p38γ is positively correlated to the differentiation grade of glioma	Human	55
CTCL	High level of p38γ is related to CTCL and is fundamental for viability of cancer	Human	4, 57
OS	p38γ may play an indispensable part in the proliferation, migration and invasion of OS cells	Human	58, 36
RCC	Overexpression of p38γ is capable to encourage RCC cell development, proliferation and migration	Human	35, 60
	The expression of p38γ mRNA and protein in RCC tissues are higher than those in normal tissues adjacent to the carcinoma	Human	35
	High p38γ levels are detected in FeNTA-induced renal cancer development and maintenance	Human	60
NSCLC	Compared with the control group, the markedly elevated level of p38γ is related to the germination of NSCLC disease	Human	61
Bladder cancer	The outcome of patients with high expression of p38γ is worse	Human	62
**Tumors suppressed by p38γ signaling**			
NSCLC	In xenograft models, p38γ is able to lessen the tumor initiation ability and tumor growth of NSCLC cells	Mouse	12

**Abbreviations**: BC, breast cancer; TNBC, triple-negative breast cancer; SCC, squamous cell cancer; HNSCC, head and neck squamous cell carcinoma; ESSC, esophageal squamous cell carcinoma; CRC, colorectal cancer; HCC, hepatocellular carcinoma; PDAC, pancreatic ductal adenocarcinoma; RCC, renal cell carcinoma; CTCL, cutaneous t-cell lymphoma; OS, osteosarcoma; NSCLC, non-small cell lung cancer; GBM, glioblastoma; WT, wild type; IEC, intestinal epithelial cell; PFD, pirfenidone; AOM/DSS, azomethane/dextran sodium sulfate; DEN, diethylnitrosamine.

## References

[1] Mertens S, Craxton M, Goedert M (1996). SAP kinase-3, a new member of the family of mammalian stress-activated protein kinases. FEBS Lett.

[2] Qi X, Pohl N M, Loesch M, Hou S, Li R, Qin J Z (2007). p38alpha antagonizes p38gamma activity through c-Jun-dependent ubiquitin-proteasome pathways in regulating Ras transformation and stress response. J Biol Chem.

[3] Loesch M, Zhi H Y, Hou S W, Qi X M, Li R S, Basir Z (2010). p38gamma MAPK cooperates with c-Jun in trans-activating matrix metalloproteinase 9. J Biol Chem.

[4] Zhang X H, Nam S, Wu J, Chen C H, Liu X, Li H (2018). Multi-Kinase Inhibitor with Anti-p38gamma Activity in Cutaneous T-Cell Lymphoma. J Invest Dermatol.

[5] Cuadrado A, Nebreda A R (2010). Mechanisms and functions of p38 MAPK signalling. Biochem J.

[6] Genera M, Samson D, Raynal B, Haouz A, Baron B, Simenel C (2019). Structural and functional characterization of the PDZ domain of the human phosphatase PTPN3 and its interaction with the human papillomavirus E6 oncoprotein. Sci Rep.

[7] Sabio G, Cerezo-Guisado M I, Del Reino P, Inesta-Vaquera F A, Rousseau S, Arthur J S (2010). p38gamma regulates interaction of nuclear PSF and RNA with the tumour-suppressor hDlg in response to osmotic shock. J Cell Sci.

[8] Escos A, Risco A, Alsina-Beauchamp D, Cuenda A (2016). p38gamma and p38delta Mitogen Activated Protein Kinases (MAPKs), New Stars in the MAPK Galaxy. Front Cell Dev Biol.

[9] Qi X, Hou S, Lepp A, Li R, Basir Z, Lou Z (2011). Phosphorylation and stabilization of topoisomerase IIalpha protein by p38gamma mitogen-activated protein kinase sensitize breast cancer cells to its poisons. J Biol Chem.

[10] Tang J, Qi X, Mercola D, Han J, Chen G (2005). Essential role of p38gamma in K-Ras transformation independent of phosphorylation. J Biol Chem.

[11] Qi X, Tang J, Loesch M, Pohl N, Alkan S, Chen G (2006). p38gamma mitogen-activated protein kinase integrates signaling crosstalk between Ras and estrogen receptor to increase breast cancer invasion. Cancer Res.

[12] Fang Y, Wang J, Wang G, Zhou C, Wang P, Zhao S (2017). Inactivation of p38 MAPK contributes to stem cell-like properties of non-small cell lung cancer. Oncotarget.

[13] Degirmenci U, Wang M, Hu J (2020). Targeting Aberrant RAS/RAF/MEK/ERK Signaling for Cancer Therapy. Cells.

[14] Cargnello M, Roux P P (2011). Activation and function of the MAPKs and their substrates, the MAPK-activated protein kinases. Microbiol Mol Biol Rev.

[15] Ono K, Han J (2000). The p38 signal transduction pathway: activation and function. Cell Signal.

[16] Wang F, Qi X M, Wertz R, Mortensen M, Hagen C, Evans J (2020). p38gamma MAPK Is Essential for Aerobic Glycolysis and Pancreatic Tumorigenesis. Cancer Res.

[17] Han J, Lee J D, Bibbs L, Ulevitch R J (1994). A MAP kinase targeted by endotoxin and hyperosmolarity in mammalian cells. Science.

[18] Cuenda A, Rousseau S (2007). p38 MAP-kinases pathway regulation, function and role in human diseases. Biochim Biophys Acta.

[19] Meng F, Wu G (2013). Is p38gamma MAPK a metastasis-promoting gene or an oncogenic property-maintaining gene?. Cell Cycle.

[20] Sahu V, Nigam L, Agnihotri V, Gupta A, Shekhar S, Subbarao N (2019). Diagnostic Significance of p38 Isoforms (p38alpha, p38beta, p38gamma, p38delta) in Head and Neck Squamous Cell Carcinoma: Comparative Serum Level Evaluation and Design of Novel Peptide Inhibitor Targeting the Same. Cancer Res Treat.

[21] Zur R, Garcia-Ibanez L, Nunez-Buiza A, Aparicio N, Liappas G, Escos A (2015). Combined deletion of p38gamma and p38delta reduces skin inflammation and protects from carcinogenesis. Oncotarget.

[22] Risco A, Cuenda A (2012). New Insights into the p38gamma and p38delta MAPK Pathways. J Signal Transduct.

[23] Kastan M B, Bartek J (2004). Cell-cycle checkpoints and cancer. Nature.

[24] Chen X, Du Y, Liu Y, He Y, Zhang G, Yang C (2018). Hyaluronan arrests human breast cancer cell growth by prolonging the G0/G1 phase of the cell cycle. Acta Biochim Biophys Sin (Shanghai).

[25] Gubern A, Joaquin M, Marques M, Maseres P, Garcia-Garcia J, Amat R (2016). The N-Terminal Phosphorylation of RB by p38 Bypasses Its Inactivation by CDKs and Prevents Proliferation in Cancer Cells. Mol Cell.

[26] Jin S, Tong T, Fan W, Fan F, Antinore M J, Zhu X (2002). GADD45-induced cell cycle G2-M arrest associates with altered subcellular distribution of cyclin B1 and is independent of p38 kinase activity. Oncogene.

[27] Liu Y, Chang Y, Cai Y (2020). circTNFRSF21, a newly identified circular RNA promotes endometrial carcinoma pathogenesis through regulating miR-1227-MAPK13/ATF2 axis. Aging (Albany NY).

[28] Conrad P W, Rust R T, Han J, Millhorn D E, Beitner-Johnson D (1999). Selective activation of p38alpha and p38gamma by hypoxia. Role in regulation of cyclin D1 by hypoxia in PC12 cells. J Biol Chem.

[29] Wang X, McGowan C H, Zhao M, He L, Downey J S, Fearns C (2000). Involvement of the MKK6-p38gamma cascade in gamma-radiation-induced cell cycle arrest. Mol Cell Biol.

[30] Perdiguero E, Pillaire M J, Bodart J F, Hennersdorf F, Frodin M, Duesbery N S (2003). Xp38gamma/SAPK3 promotes meiotic G(2)/M transition in Xenopus oocytes and activates Cdc25C. EMBO J.

[31] Tomas-Loba A, Manieri E, Gonzalez-Teran B, Mora A, Leiva-Vega L, Santamans A M (2019). p38gamma is essential for cell cycle progression and liver tumorigenesis. Nature.

[32] Meng F, Zhang H, Liu G, Kreike B, Chen W, Sethi S (2011). p38gamma mitogen-activated protein kinase contributes to oncogenic properties maintenance and resistance to poly (ADP-ribose)-polymerase-1 inhibition in breast cancer. Neoplasia.

[33] Kukkonen-Macchi A, Sicora O, Kaczynska K, Oetken-Lindholm C, Pouwels J, Laine L (2011). Loss of p38gamma MAPK induces pleiotropic mitotic defects and massive cell death. J Cell Sci.

[34] Su C, Sun Q, Liu S, Wang H, Feng L, Cao Y (2019). Targeting p38gamma to inhibit human colorectal cancer cell progression. Biochem Biophys Res Commun.

[35] Chen X F, Pan Y S, Zheng B, Lu Q (2019). p38gamma overexpression promotes renal cell carcinoma cell growth, proliferation and migration. Biochem Biophys Res Commun.

[36] Shi C, Cheng W N, Wang Y, Li D Z, Zhou L N, Zhu Y C (2020). p38gamma overexpression promotes osteosarcoma cell progression. Aging (Albany NY).

[37] Zhang D, Li Y, Wang R, Li Y, Shi P, Kan Z (2016). Inhibition of REST Suppresses Proliferation and Migration in Glioblastoma Cells. Int J Mol Sci.

[38] Qi X, Zhi H, Lepp A, Wang P, Huang J, Basir Z (2012). p38gamma mitogen-activated protein kinase (MAPK) confers breast cancer hormone sensitivity by switching estrogen receptor (ER) signaling from classical to nonclassical pathway via stimulating ER phosphorylation and c-Jun transcription. J Biol Chem.

[39] Suresh P S, Ma S, Migliaccio A, Chen G (2014). Protein-tyrosine phosphatase H1 increases breast cancer sensitivity to antiestrogens by dephosphorylating estrogen receptor at Tyr537. Mol Cancer Ther.

[40] Chen H, Wang X, Guo F, Li P, Peng D, He J (2019). Impact of p38gamma mitogen-activated protein kinase (MAPK) on MDA-MB-231 breast cancer cells using metabolomic approach. Int J Biochem Cell Biol.

[41] Rosenthal D T, Iyer H, Escudero S, Bao L, Wu Z, Ventura A C (2011). p38gamma promotes breast cancer cell motility and metastasis through regulation of RhoC GTPase, cytoskeletal architecture, and a novel leading edge behavior. Cancer Res.

[42] Xu M, Wang S, Ren Z, Frank J A, Yang X H, Zhang Z (2016). Chronic ethanol exposure enhances the aggressiveness of breast cancer: the role of p38gamma. Oncotarget.

[43] Xu M, Ren Z, Wang X, Comer A, Frank J A, Ke Z J (2016). ErbB2 and p38gamma MAPK mediate alcohol-induced increase in breast cancer stem cells and metastasis. Mol Cancer.

[44] Dongre A, Rashidian M, Reinhardt F, Bagnato A, Keckesova Z, Ploegh H L (2017). Epithelial-to-Mesenchymal Transition Contributes to Immunosuppression in Breast Carcinomas. Cancer Res.

[45] Xu M, Wang S, Wang Y, Wu H, Frank J A, Zhang Z (2018). Role of p38gamma MAPK in regulation of EMT and cancer stem cells. Biochim Biophys Acta Mol Basis Dis.

[46] Kim C, Gao R, Sei E, Brandt R, Hartman J, Hatschek T (2018). Chemoresistance Evolution in Triple-Negative Breast Cancer Delineated by Single-Cell Sequencing. Cell.

[47] Tam W L, Lu H, Buikhuisen J, Soh B S, Lim E, Reinhardt F (2013). Protein kinase C alpha is a central signaling node and therapeutic target for breast cancer stem cells. Cancer Cell.

[48] Qi X, Yin N, Ma S, Lepp A, Tang J, Jing W (2015). p38gamma MAPK Is a Therapeutic Target for Triple-Negative Breast Cancer by Stimulation of Cancer Stem-Like Cell Expansion. Stem Cells.

[49] Zheng S, Yang C, Liu T, Liu Q, Dai F, Sheyhidin I (2016). Clinicopathological significance of p38beta, p38gamma, and p38delta and its biological roles in esophageal squamous cell carcinoma. Tumour Biol.

[50] Gonzalez-Teran B, Matesanz N, Nikolic I, Verdugo M A, Sreeramkumar V, Hernandez-Cosido L (2016). p38gamma and p38delta reprogram liver metabolism by modulating neutrophil infiltration. EMBO J.

[51] Yin N, Qi X, Tsai S, Lu Y, Basir Z, Oshima K (2016). p38gamma MAPK is required for inflammation-associated colon tumorigenesis. Oncogene.

[52] Hou S, Suresh P S, Qi X, Lepp A, Mirza S P, Chen G (2012). p38gamma Mitogen-activated protein kinase signals through phosphorylating its phosphatase PTPH1 in regulating ras protein oncogenesis and stress response. J Biol Chem.

[53] Hou S W, Zhi H Y, Pohl N, Loesch M, Qi X M, Li R S (2010). PTPH1 dephosphorylates and cooperates with p38gamma MAPK to increase ras oncogenesis through PDZ-mediated interaction. Cancer Res.

[54] Icard P, Fournel L, Wu Z, Alifano M, Lincet H (2019). Interconnection between Metabolism and Cell Cycle in Cancer. Trends Biochem Sci.

[55] Yang K, Liu Y, Liu Z, Liu J, Liu X, Chen X (2013). p38gamma overexpression in gliomas and its role in proliferation and apoptosis. Sci Rep.

[56] Bi J, Chowdhry S, Wu S, Zhang W, Masui K, Mischel P S (2020). Altered cellular metabolism in gliomas - an emerging landscape of actionable co-dependency targets. Nat Rev Cancer.

[57] Haller V, Nahidino P, Forster M, Laufer S A (2020). An updated patent review of p38 MAP kinase inhibitors (2014-2019). Expert Opin Ther Pat.

[58] Cui C, Shi X (2017). miR-187 inhibits tumor growth and invasion by directly targeting MAPK12 in osteosarcoma. Exp Ther Med.

[59] Cheng L, Wang C, Jing J (2016). Cell Cycle Kinases in Osteosarcoma: Potential for Therapeutic Intervention. Curr Pharm Des.

[60] Aguilar-Alonso F A, Solano J D, Vargas-Olvera C Y, Pacheco-Bernal I, Pariente-Perez T O, Ibarra-Rubio M E (2015). MAPKs' status at early stages of renal carcinogenesis and tumors induced by ferric nitrilotriacetate. Mol Cell Biochem.

[61] Sahu V, Mohan A, Dey S (2019). p38 MAP kinases: plausible diagnostic and prognostic serum protein marker of non-small cell lung cancer. Exp Mol Pathol.

[62] Lin J, Yang J, Xu X, Wang Y, Yu M, Zhu Y (2020). A robust 11-genes prognostic model can predict overall survival in bladder cancer patients based on five cohorts. Cancer Cell Int.

[63] Davis A P, Grondin C J, Johnson R J, Sciaky D, King B L, McMorran R (2017). The Comparative Toxicogenomics Database: update 2017. Nucleic Acids Res.

[64] Yamaguchi R, Kawata J, Yamamoto T, Ishimaru Y, Sakamoto A, Ono T (2015). Mechanism of interferon-gamma production by monocytes stimulated with myeloperoxidase and neutrophil extracellular traps. Blood Cells Mol Dis.

[65] Wishart D S, Feunang Y D, Guo A C, Lo E J, Marcu A, Grant J R (2018). DrugBank 5.0: a major update to the DrugBank database for 2018. Nucleic Acids Res.

[66] Gaines S, van Praagh J B, Williamson A J, Jacobson R A, Hyoju S, Zaborin A (2020). Western Diet Promotes Intestinal Colonization by Collagenolytic Microbes and promotes Tumor Formation after Colorectal Surgery. Gastroenterology.

[67] Dempsey J, Zhang A, Cui J Y (2018). Coordinate regulation of long non-coding RNAs and protein-coding genes in germ-free mice. BMC Genomics.

[68] Koh A, Molinaro A, Stahlman M, Khan M T, Schmidt C, Manneras-Holm L (2018). Microbially Produced Imidazole Propionate Impairs Insulin Signaling through mTORC1. Cell.

[69] Moscat J, Diaz-Meco M T (2009). p62 at the crossroads of autophagy, apoptosis, and cancer. Cell.

[70] Koh A, Manneras-Holm L, Yunn N O, Nilsson P M, Ryu S H, Molinaro A (2020). Microbial Imidazole Propionate Affects Responses to Metformin through p38gamma-Dependent Inhibitory AMPK Phosphorylation. Cell Metab.

[71] Morales D R, Morris A D (2015). Metformin in cancer treatment and prevention. Annu Rev Med.

[72] Xiao-Jie L, Hui-Ying X, Zun-Ping K, Jin-Lian C, Li-Juan J (2015). CRISPR-Cas9: a new and promising player in gene therapy. J Med Genet.

[73] Vojta A, Dobrinic P, Tadic V, Bockor L, Korac P, Julg B (2016). Repurposing the CRISPR-Cas9 system for targeted DNA methylation. Nucleic Acids Res.

[74] Hilton I B, D'Ippolito A M, Vockley C M, Thakore P I, Crawford G E, Reddy T E (2015). Epigenome editing by a CRISPR-Cas9-based acetyltransferase activates genes from promoters and enhancers. Nat Biotechnol.

[75] Thakore P I, D'Ippolito A M, Song L, Safi A, Shivakumar N K, Kabadi A M (2015). Highly specific epigenome editing by CRISPR-Cas9 repressors for silencing of distal regulatory elements. Nat Methods.

[76] Doroshow D B, Bhalla S, Beasley M B, Sholl L M, Kerr K M, Gnjatic S (2021). PD-L1 as a biomarker of response to immune-checkpoint inhibitors. Nat Rev Clin Oncol.

[77] Risco A, Martin-Serrano M A, Barber D F, Cuenda A (2018). p38gamma and p38delta Are Involved in T Lymphocyte Development. Front Immunol.

